# Senescence Is the Main Trait Induced by Temozolomide in Glioblastoma Cells

**DOI:** 10.3390/cancers14092233

**Published:** 2022-04-29

**Authors:** Lea Beltzig, Christian Schwarzenbach, Petra Leukel, Katrin B. M. Frauenknecht, Clemens Sommer, Alessandro Tancredi, Monika E. Hegi, Markus Christmann, Bernd Kaina

**Affiliations:** 1 Institute of Toxicology, University Medical Center, 55131 Mainz, Germany; lea.beltzig@uni-mainz.de (L.B.); schwarzenbach@uni-mainz.de (C.S.); mchristm@uni-mainz.de (M.C.); 2 Institute of Neuropathology, University Medical Center, 55131 Mainz, Germany; petra.leukel@unimedizin-mainz.de (P.L.); katrin.frauenknecht@lns.etat.lu (K.B.M.F.); clemens.sommer@unimedizin-mainz.de (C.S.); 3 Neuroscience Research Center and Neurosurgery, Lausanne University Hospital, H-1066 Epalinges, Switzerland; alessandro.tancredi@chuv.ch (A.T.); monika.hegi@chuv.ch (M.E.H.)

**Keywords:** temozolomide, apoptosis, senescence, glioblastoma, O^6^-methylguanine, MGMT

## Abstract

**Simple Summary:**

Temozolomide (TMZ) is the first-line drug in the treatment of glioblastoma. Due to the formation of the DNA damage O^6^-methylguanine, it is toxic to cancer cells, resulting in the induction of apoptosis. However, the damage also induces cellular senescence. Here, we show that not apoptosis, but cellular senescence is the main response triggered by TMZ-induced DNA damage. We analyzed the senescent cells in detail and show that they are characterized by a high level of non-repaired DNA double-strand breaks that trigger the DNA damage response. The primary damage, O^6^-methylguanine, is the upstream trigger, but it is not required for maintaining the senescent state. Cells which acquired temozolomide resistance also became resistant to the induction of senescence. Comparing specimens from primary and recurrent glioblastoma, we show that recurrences contain a higher proportion of senescent cells than the primary tumor, indicating that induction of senescence also occurs upon treatment in vivo, which likely plays a role in therapy resistance.

**Abstract:**

First-line drug in the treatment of glioblastoma, the most severe brain cancer, is temozolomide (TMZ), a DNA-methylating agent that induces the critical damage O^6^-methylguanine (O^6^MeG). This lesion is cytotoxic through the generation of mismatch repair-mediated DNA double-strand breaks (DSBs), which trigger apoptotic pathways. Previously, we showed that O^6^MeG also induces cellular senescence (CSEN). Here, we show that TMZ-induced CSEN is a late response which has similar kinetics to apoptosis, but at a fourfold higher level. CSEN cells show a high amount of DSBs, which are located outside of telomeres, a high level of ROS and oxidized DNA damage (8-oxo-guanine), and sustained activation of the DNA damage response and histone methylation. Despite the presence of DSBs, CSEN cells are capable of repairing radiation-induced DSBs. Glioblastoma cells that acquired resistance to TMZ became simultaneously resistant to TMZ-induced CSEN. Using a Tet-On glioblastoma cell system, we show that upregulation of MGMT immediately after TMZ completely abrogated apoptosis and CSEN, while induction of MGMT long-term (>72 h) after TMZ did not reduce apoptosis and CSEN. Furthermore, upregulation of MGMT in the senescent cell population had no impact on the survival of senescent cells, indicating that O^6^MeG is required for induction, but not for maintenance of the senescent state. We further show that, in recurrent GBM specimens, a significantly higher level of DSBs and CSEN-associated histone H3K27me3 was observed than in the corresponding primary tumors. Overall, the data indicate that CSEN is a key node induced in GBM following chemotherapy.

## 1. Introduction

The first-line therapeutic for high-grade gliomas, such as WHO grade 4 classified glioblastoma (GBM), is the DNA-methylating agent temozolomide (TMZ) [1,2]. After oral administration, TMZ spontaneously decomposes, yielding 3-methyl-(triazen-1-yl) imidazole-4-carboxamide (MTIC), which is further degraded to 5-aminoimidazole-4-carboxamide (AIC) and a monomethylhydrazine. In an S_N_1 alkylating process, the reactive species methylates the nuclear DNA at various sites, yielding N- and O-methylation products such as N7-methylguanine, N3-methylguanine, N3-methyladenine, and O^6^-methylguanine (O^6^MeG) [3,4,5]. Although O^6^MeG accounts for only ~7% of the adducts induced by TMZ, it is the main mutagenic, carcinogenic, genotoxic, and cytotoxic lesion responsible for the induction of autophagy, senescence, and apoptosis [6,7].

The O^6^MeG lesion is subject to repair by the O^6^-methylguanine-DNA methyltransferase (MGMT) suicide enzyme [8]. MGMT promoter hypermethylation leads to downregulation of MGMT expression; it is present in about 40% of glioma grade 4 cases, and about 17% of GBM show complete absence of MGMT activity [9]. Since MGMT activity is necessary for O^6^MeG repair, promoter-methylated GBM shows a better response to TMZ than unmethylated GBM [10,11,12]. If cells lack MGMT, O^6^MeG persists in the tumor cell DNA, which leads to mispairing with thymine and activation of the mismatch repair (MMR) system. Due to the high affinity of thymine to O^6^MeG, thymine is reinserted opposite O^6^MeG during MMR, causing futile MMR cycles. The widely accepted model of DSB formation following TMZ treatment claims that these futile MMR cycles lead to blockage in the second round of DNA replication following damage induction and the formation of DNA double-strand breaks (DSBs) [13]. Time-course experiments measuring apoptosis and DSB formation, as well as results obtained with synchronized cells, confirmed this model [14,15]. Further experiments revealed that the DSBs trigger the DNA damage response (DDR) pathway, starting with the activation of ATR, followed by ATM and their downstream targets CHK1 and CHK2, as well as the HIPK2–p53 axis [16,17]. Eventually, O^6^MeG triggers apoptosis and cellular senescence in glioblastoma cells [7,18,19].

In low dose ranges that are sufficient to induce cytotoxicity through O^6^MeG in MGMT lacking cells, N-alkylations are repaired by base excision repair (BER) [6]. One could claim that BER can be saturated through higher TMZ dosages, and then N-alkylated DNA lesions might also contribute to DSB formation and cell death [20]. However, it is unlikely to achieve cell death resulting from N-alkylations neither in MGMT proficient nor deficient tumors in a therapeutic setting, since the therapeutically relevant doses tolerable for patients are too low. The minor lesion O^6^MeG is, therefore, the key adduct in inducing toxic cellular responses following TMZ treatment of patients.

Standard therapy for GBM relies on maximal safe resection of the tumor, followed by concomitant treatment with radiation and TMZ, followed by TMZ monotherapy [21]. Furthermore, in recurrent therapy, TMZ is often used in daily doses over long periods of time, which is sufficiently tolerable by the patients [22]. Despite these efforts, GBM patients have a dismal prognosis due to the weak therapeutic efficiency of TMZ [2]. A better understanding of the anticancer mechanisms of TMZ is, therefore, highly desirable. Accordingly, it is important to identify the main cellular responses induced by TMZ. Is cell death the main trait induced by TMZ, or does TMZ induce mainly cytostatic effects? To address this question, we measured the amount of apoptosis and cellular senescence (CSEN), as well as respective molecular markers in GBM cells after TMZ treatment. Time-course experiments revealed that CSEN is induced early after TMZ treatment, increasing by up to 80% over time, clarifying that CSEN is the main pathway induced by TMZ in cells in vitro. Apoptosis is induced simultaneously in much lower percentages. We characterized the TMZ-induced senescent cells and show that they display high levels of DSBs and exhibit a sustained DDR. Staining of patient-derived GBM samples indicated that this is true not only in vitro, but also in a clinical setup. Overall, our data revealed that CSEN is the main trait induced by TMZ in GBM cells.

## 2. Materials and Methods

### 2.1. Cell Lines and Culture Conditions

The human glioblastoma cell lines LN229 and A172 were purchased from the American Type Culture Collection (ATCC) and were described in terms of TMZ responses in a previous publication [23]. The LN229 MGMT-inducible cell line (clone C12) was generated by stable lentiviral transfection of LN229 cells with MGMT cDNA [24] cloned into the tetracycline (tet)-inducible vector pCW22. In brief, the recipient vector pCW22-Cas9, received from Joachim Ligner, Lausanne, Switzerland [25], was restricted with *Sal*I and *Sbf*I (New England Biolabs, MA, USA) to remove the Cas9 gene (4 kb) from the trAT (Tet-On)-containing plasmid (9.6 kb). The insert pSV2MGMT was amplified and PCR-cloned to add restriction sites *Sal*I and *Sbf*I. Isolated, PCR-cloned MGMT cDNA was digested with *Sal*I and *Sbf*I to generate compatible ends. The restricted insert and the pCW22 recipient vector were ligated to obtain the doxycycline-inducible pCW22-MGMT vector. Lentiviral vector production was performed in HEK293T cells using the packaging vector pCMV9.74 (Addgene, 22036, Watertown, MA, USA) and the envelope vector pMD2.G (Addgene, 12259), followed by infection/transduction of LN229 using standard procedures. Transduced cells were selected with blasticidin (#R21001; ThermoFisher, MA, USA) treatment. Single-cell clones were obtained and tested for inducibility and leakiness, and clone LN229-MGMTind_C12 was selected for experiments (herein briefly designated as LN229-MGMT^ind^). All cells were cultured in DMEM containing Glutamax (Gibco, Life Technologies Corporation, Paisley, UK) and 10% fetal calf serum. Cells were kept at 37 °C in a humidified 5% CO_2_ atmosphere. To ensure exponential growth at the timepoint of treatment, cells were seeded 48 h before treatment for all experiments. Cells were seeded in six-well dishes with 3 mL of medium; 1 × 10^5^ cells were seeded per dish.

### 2.2. Drugs and Treatments

TMZ was obtained from Dr Geoff Margison (University of Manchester, Manchester, UK). To create a 150 mM stock solution, it was dissolved in DMSO and stored in batches at −80 °C until use. Immediately before use, TMZ was diluted in sterile distilled water to a 15 mM working solution, which was used only once. Diluted TMZ was added to the cell culture medium to gain the desired final concentration of 50 µM. The amount of DMSO in the medium did not exceed 0.05%, and the control showed no cytotoxic effect at this concentration. Doxycycline (Sigma-Aldrich, Steinheim, Germany) was prepared in sterile deionized water at a 50 µg/mL stock concentration, aliquoted, and stored at −20 °C until use. Aliquots were brought to room temperature immediately before use, and doxycycline was added directly to the medium to reach the final concentration of 100 ng/mL, necessary to induce MGMT. Cells were irradiated with 6 Gy using a Gammacell Irradiator 2000 (Cs-137 source, Molsgaard Medical, Denmark). For repair experiments, cells were kept in their respective dishes and medium during irradiation. Cells that were measured immediately after irradiation were collected and measured in Eppendorf tubes, and directly stored on ice.

### 2.3. Quantification of Apoptosis and Necrosis

Flow cytometry measurement of FITC-coupled annexin V (AV/FITC) (Miltenyi Biotec GmbH, Bergisch GLadbach, Germany) and propidium iodide (PI) staining of the cells was used to determine the fraction of apoptotic and necrotic cells. Therefore, cells including the supernatant were collected in 15 mL falcon tubes and labeled with 2.5 µL of AV/FITC (Miltenyi Biotec GmbH) in 50 µL of 1× annexin-binding buffer at RT for 15 min in the dark. From this point on, cells were kept in the dark until measurement. For PI staining, 10 µL of a 50 µg/mL PI stock solution (Sigma-Aldrich, Steinheim, Germany) and additional annexin-binding buffer were added to each sample, and cells were incubated for 10 min on ice. The FACS Canto II flow cytometer (Becton Dickinson GmbH, Heidelberg, Germany) was used for data acquisition, while the Flowing Software 2.5.1 program (Perttu Terho, Turku Center for Biotechnology, Turku, Finland) was used for data analysis. Proliferating cells served as control. Annexin V^−^/PI^−^ cells were defined as living, annexin V^+^/PI^−^ cells were defined as apoptotic cells, and annexin V^+^/PI^+^ cells were defined as necrotic cells.

### 2.4. Quantification of Cellular Senescence

The amount of SA-β-galactosidase, a marker for senescence, was used to determine the percentage of senescent cells within the population. Two staining methods were used, X-Gal and C12FDG. For C12FDG, cells were preincubated with 300 µM chloroquine (Sigma-Aldrich) for 30 min in the incubator, to inhibit endogenous β-galactosidase activity, and kept in the dark from this point on. C12FDG (Abcam) was added from a 20 mM stock solution to a final concentration of 33 µM to each sample, and cells were incubated for an additional 90 min in the incubator. Cells were then collected, washed, resuspended in an adjusted amount of PBS, and stored on ice until measurement. The FACS Canto II flow cytometer (Becton Dickinson GmbH, Heidelberg, Germany) was used for data acquisition, while the Flowing Software 2.5.1 program (Perttu Terho, Turku Center for Biotechnology, Turku, Finland) was used for data analysis. Proliferating cells served as control; cells showing a stronger fluorescence signal than proliferating cells were defined as senescent. For the X-gal staining, cells were washed with cold PBS and fixed for 5 min at RT (2% formaldehyde and 0.2% glutaraldehyde in PBS). Cells were washed again with cold PBS and stained with freshly prepared X-gal staining buffer (30 mM citric acid, 100 mM Na_2_PO_4_·12H_2_O, 125 mM NaCl, 1.6 mM MgCl_2_, 4.2 mM potassium ferricyanide and ferrocyanide, and 0.03% X-gal pH 6) overnight in the incubator without CO_2_. Before analysis, cells were washed again with PBS. X-gal-positive cells were quantified using an inverse microscope and the ImageJ software (Wayne Rasband, NIH).

### 2.5. Quantification of Polyploidy

For the quantification of polyploid cells within the population, attached cells were harvested and transferred into 15 mL falcon tubes, washed with cold PBS, and fixed in 70% ice-cold ethanol. Cells were the incubated with 1 µL/mL RNaseA (Sigma-Aldrich, Karlsruhe, Germany) for 1 h at RT to reduce the RNA content. For analysis, PI (Sigma-Aldrich, Karlsruhe, Germany) was added to each sample, and cells were incubated for 10–15 min. The polyploid population was measured using the FACS Canto II flow cytometer (Becton Dickinson GmbH, Heidelberg, Germany), and the data were analyzed using the Flowing Software 2.5.1 program (Perttu Terho, Turku Center for Biotechnology, University of Turku, Finland). Proliferating cells served as control.

### 2.6. Quantification of γH2AX, 53BP1, and Trf1 Foci

The foci assay for γH2AX, 53BP1, and Trf1 was performed as previously described [26]. Briefly, cells grown on coverslips were fixed, blocked, and stained for γH2AX and 53BP1 or γH2AX and Trf1. Foci for γH2AX and 53BP1 were assessed by LSM using a single focus plane; for γH2AX and Trf1 quantification, the whole cell was screened, and images were stacked. For each experiment, at least 100 cells were analyzed. Primary antibodies used were γH2AX (1:500, rabbit, Cell Signaling Technology, MA, USA, mAb #9718S), γH2AX (1:500, mouse, Merck Millipore, Darmstadt, Germany, 05-636), 53BP1 (1:500, mouse, Sigma Aldrich, Karlsruhe, Germany, MAB 3802), and Trf1 (1:500, mouse, Abcam, Cambridge, UK, ab10579). Secondary antibodies used were Alexa Fluor 488 goat anti-mouse (1:1000, Thermo Fisher Scientific Invitrogen, Waltham, USA, A11017) and Cy3 goat anti-rabbit (1:1000, Abcam, ab97075). The software ImageJ (Wayne Rasband, NIH, Bethesda, MD, USA) was used to count foci and measure colocalization.

### 2.7. Neutral and FPG-Alkaline Comet Assays

The neutral and FPG-alkaline comet assays were performed to measure the number of SSBs, number of DSBs, and amount of oxidative stress induced by TMZ. treatment. Comet assays were performed as previously described [27]. Briefly, cells were collected and embedded in ultralow-melting-point agarose. They were then lysed, and the DNA was denatured. In case of the FPG-alkaline comet assay, cells were preincubated with FPG. Lysed and denatured cells were then subject to single-cell electrophoresis, after which slides were fixed in ethanol and dried. For analysis, slides were incubated with PI and measured using a fluorescence microscope (Microphot-FXA, Nikon, Tokyo, Japan) and the Comet IV software (Perceptive Imaging, Liverpool, UK). Untreated proliferating cells served as a negative control, while proliferating cells treated with 300 µM tBOOH (Sigma-Aldrich, Karlsruhe, Germany) for 30 min served as a positive control.

### 2.8. Protein Extraction and Western Blot Analysis

Protein was extracted from whole cells using the cell extraction buffer from Invitrogen (Invitrogen Corporation, Camarillo, C) and the respective protocol. Cell lysates were mixed with RotiLoad 1 (Carl Roth GmbH + Co. KG, Karlsruhe, Germany), boiled for 5–10 min at 95 °C, and stored at −20 °C until use. For WB analysis, 10–100 µg of protein was loaded onto a 5%, 10%, or 15% acrylamide gel and run at 120 mA for 1.5–2 h. Separated proteins were transferred to a 0.2 μm Protean nitrocellulose membrane as described [28]. To avoid unspecific binding, membranes were incubated in PBS containing 5% BSA for 1 h at RT. Proteins were detected by means of Odyssee. The primary antibodies were pATMSer1981 (1:1000, ms mAb, Cell Signaling, Cambridge, UK, #4526) pATRThr1989 (1:1000, rb pAb, Invitrogen #PA77873), pChk1S345 (1:1000, rb mAb, Cell Signaling, #2348P), pChk2 (1:1000, rb mAB, Cell Signaling, #2661), Nrf2 (1:1000, mouse mAb, Santa Cruz, Dallas, USA, #sc365949), HO1 (1:1000, ms mAbm Santa Cruz, #sc136960), HSP90 (1:5000, ms mAb, Santa Cruz, #sc13119), MGMT (1:750, rb pAb, from B. Kaina, 9-Max), p53 (1:1000, ms mAb, Sigma-Aldrich, Karlsruhe, Germany, #OP09), p53ser15 (1:1000, rb pAb, Cell Signalling, #9284S), p53ser46 (1:1000, ms mAb, BD, Heidelberg, Germany, #558245), p21 (1:1000, rb mAb, Cell Signaling, #2947S), H3K27me3 (1:1000, rb mAb, Cell Signaling, #9733S), H3K9me3 (1:1000, rb mAb, Cell Signaling, #13969S), γH2AX (1:1000, rb mAb, Abcam, #ab81299), Bcl-2 (1:1000, ms mAB, Santa Cruz, #sc7832), Bcl-XL (1:1000, rb mAb, Cell Signaling, #2764T), Bcl-W (1:1000, rb mAb, Cell Signaling #2724S), MSH2 (1:1000, ms mAb, Abcam, #ab52266), MSH6 (1:1000, ms mAb, Abcam, #ab14204), and Rad51 (1:1000, rb pAb, Abcam, #ab63801). Secondary antibodies used were IRDye goat anti-mouse and anti-rabbit IgG 800CW and IRDye goat anti-mouse and anti-rabbit IgG 680RD (1:5000, Li-Corn Inc., Lincoln, NE, USA). Proteins were detected using the Odyssee 9120 (Li-Cor Inc., Lincoln, NE, USA).

### 2.9. Immunohistochemistry

Tumor samples were obtained from glioblastoma patients (WHO grade 4) and embedded immediately after resection in paraffin. A total of 10 paired samples of primary and recurrent tumors were studied by immunohistochemistry and TUNEL. Immunohistochemistry was performed on 4 µm thick routinely processed formalin-fixed and paraffin-embedded tissue sections. After dewaxing, antigen retrieval using EnVision FLEX Target Retrieval Solution, high pH (Dako #S2368, Glostrup, Denmark) was performed. Afterward, endogenous peroxidase was blocked by peroxidase-blocking solution (Dako, Glostrup, Denmark), and sections were stained with anti-γH2AX primary antibody (Dako, #9718S) 1:500 and anti-trimethyl-histone H3 (Lys27) primary antibody (mAb #9718S) 1:200 (Cell Signaling Technology) using an immunostainer (Dako Autostainer Plus, Dako, Glostrup, Denmark). Immunoreactivity was visualized by the universal immuno-enzyme polymer method (Nichirei Biosciences, Tokyo, Japan). Finally, sections were developed in diaminobenzidine (Lab Vision Cooperation, Fermont, CA, USA). Omission of the primary antisera in a subset of control slides resulted in no immunostaining at all. The quantification of γH2AX, TUNEL, and H3K27me3 in patient tumor samples occurred through evaluation of each slide by light microscopy (Axiovert 35, Carl Zeiss AG, Oberkochen, Germany) at 100-fold magnification. For each tumor sample, 10 different tumor areas were evaluated using the ImageJ software (Wayne Rasband, NIH, Bethesda, USA). Bars indicate the median ± SEM of percentages of positively stained cells from each picture. For immunohistochemistry with methylated histones, we used H3K9me3 (ab8898 rabbit) and H3K27me3 (ab192985 rabbit) from Abcam, Cambridge, UK.

### 2.10. Patients and Ethic Statement

This study was performed in agreement with the declaration of Helsinki on the use of human material for research. In accordance with the ethics committee of Rhineland-Palatinate, written informed consent of all patients or their custodians was obtained for “scientific use of tumor tissue not needed for histopathological diagnosis” in the admission contract of the University Medical Center Mainz (§ 14 AVB), with approval of the local ethics committee (No. 2020-15261). Tumor tissue was obtained from resected primary and recurrent glioblastoma in the Department of Neurosurgery and examined at the Institute of Neuropathology, University Medical Center, Mainz. Ten patients diagnosed with glioblastoma grade 4 were included (paired samples).

### 2.11. Statistical Analysis and Mathematical Assessments

Data are presented as the mean of at least three independent experiments ± SEM and compared by unpaired *t*-test with Welch’s correction.

## 3. Results

### 3.1. Kinetics of CSEN

**Figure 1:**
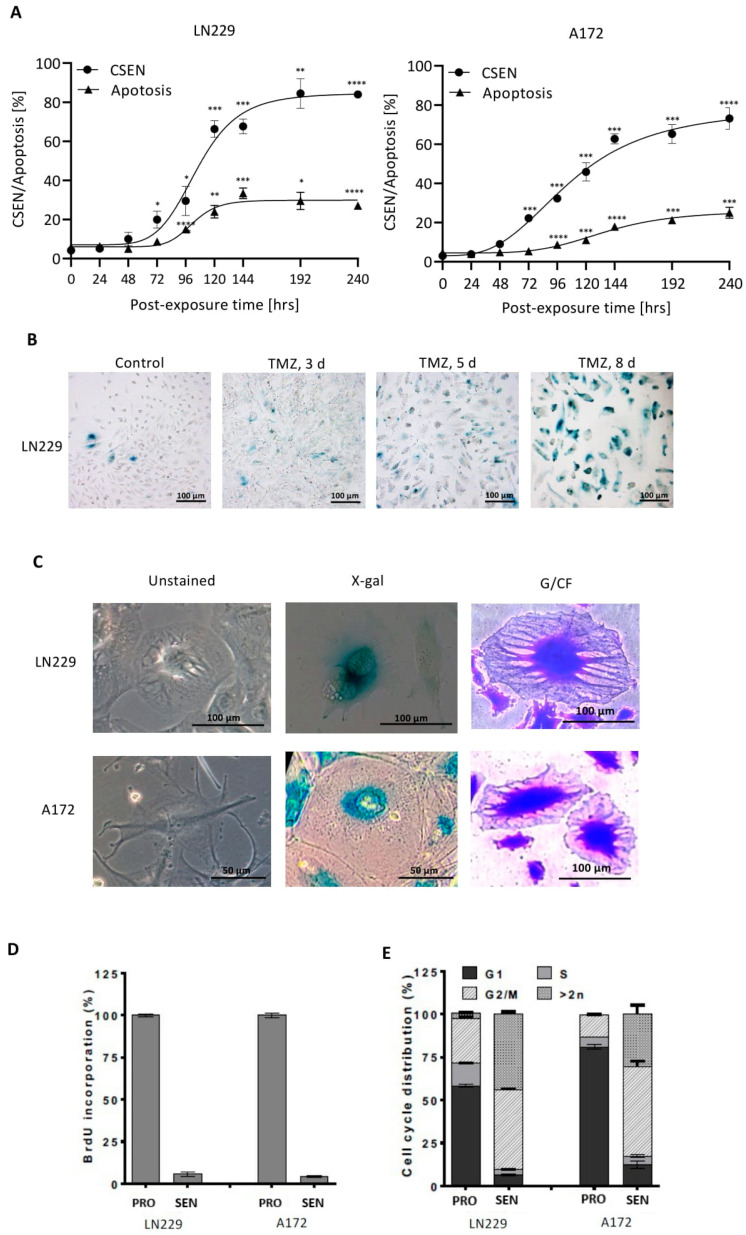
Kinetics of induction of apoptosis and CSEN in LN229 and A172 glioblastoma cells. (**A**) Kinetics of senescence and apoptosis following TMZ (50 µM treatment). Each measurement point represents the mean of at least three independent experiments, each performed in duplicate. Significance levels are indicated. * *p* < 0.05, ** *p* < 0.01, *** *p* < 0.001; **** *p* < 0.0001 (**B**) Images of LN229 cells: nontreated control after 5 days, and TMZ-treated populations 3, 5, and 8 days after addition of TMZ to the medium. (**C**) Images of senescent cells 8 days after treatment (50 µM TMZ) not stained and stained with X-Gal. Righthand site: Representative examples of senescent LN229 cells (giant cell morphology) stained with Giemsa/Carbol Fuchsin (G/CF). (**D**) DNA replication in proliferating (con) and TMZ-treated senescent population, measured 6 days after treatment. (**E**) Cell-cycle distribution of proliferating (con) and TMZ-induced senescent cells, measured 120 h after treatment.

Here, we show that apoptosis induced by TMZ in MGMT lacking GBM cells is a late response, occurring not earlier than 4 days after the onset of treatment (Figure 1A). This late response is due to the conversion of the critical lesion O^6^MeG by MMR and DNA replication into DSBs and activation of the DDR-dependent cell death pathway [29]. To elucidate whether CSEN shows similar or different kinetics, we simultaneously determined the time course of apoptosis and CSEN in MGMT-deficient LN229 and A172 cells. CSEN manifested after 3 days following TMZ and increased to a plateau value, which was reached after 6–8 days (Figure 1A and Figure S1). It is important to note that the yield of CSEN was clearly higher than apoptosis (Figure 1A), indicating that senescence is the main trait triggered by O^6^MeG adducts induced by TMZ. The quantitative data obtained by C12FDG flow cytometry were confirmed by SA-β-GAL cytochemistry, yielding similar results (Figure S2). The data revealed maximal expression of the senescence phenotype after 8 days (representative immunostainings at different times after TMZ treatment are shown in Figure 1B and Figure S1A), where about 80% of the cell population exhibited the CSEN phenotype. These cells displayed a flat morphology (Figure 1C). They were robust and viable, and they could be trypsinized and reseeded for further experimentation. They did not incorporate BrdU, i.e., they were not replicating (Figure 1D); the majority were arrested in G2, and they showed a high level of polyploidy (Figure 1E).

### 3.2. DNA Double-Strand Breaks in Senescent Cells

**Figure 2:**
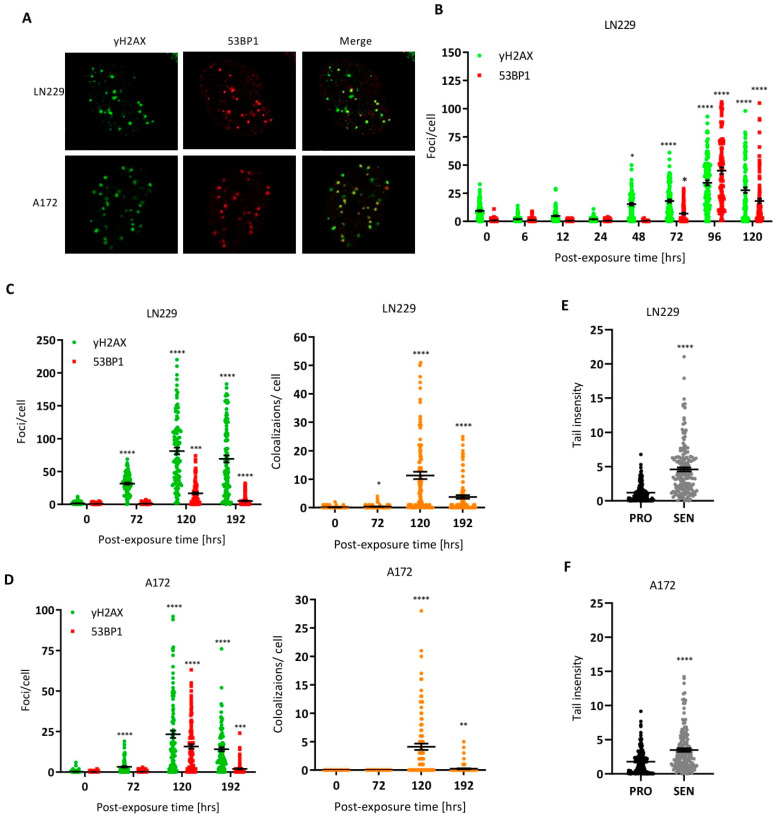
DNA double-strand breaks precede apoptosis and CSEN and persist in senescent cells. (**A**) Exemplary picture of γH2AX and 53BP1 foci staining in LN229 and A172 cells following treatment with 50 µM TMZ for 5 days. (**B**) Kinetics of DSBs measured by γH2AX and 53BP1 staining in LN229 cells following 20 µM TMZ treatment. (**C**–**F**) Sustained DSBs in LN229 (**C**,**D**) and A172 (**E**,**F**) cells following 50 µM TMZ treatment, shown by γH2AX and 53BP1 staining. Nuclear foci and colocalization of foci were determined (**C**,**D**). (**E**,**F**) DSBs as measured by the neutral comet assay in proliferating (control) and senescent cells (CSEN). * *p* < 0.05, ** *p* < 0.01, *** *p* < 0.001; **** *p* < 0.0001.

The primary killing lesion O^6^MeG gives rise to the formation of DSBs that trigger apoptosis and senescence pathways. Here, we addressed the question of whether TMZ-induced senescent cells display DSBs or whether DSBs are only transiently induced and repaired in the post-exposure period when senescent cells accumulate in the population. To this end, we determined the γH2AX and 53BP1 foci levels as a function of time after treatment with TMZ (50 µM). The colocalized foci are considered to be safe markers of DSBs (for representative immunostainings, see Figure 2A). As shown in Figure 2B, the foci number per nucleus was not enhanced up to 24 h after treatment. Only 48 h later for γH2AX, and 72 h later for γH2AX and 53BP1 did levels increase significantly above the control level. This is in line with the late induction of DSBs, which is explained on the basis of MMR-mediated processing of O^6^MeG/T mismatches [14]. Surprisingly, the level of DSBs remained very high (up to 100 foci per cell) 96 and 120 h after treatment, when the population consisted of up to 80% senescent cells. Even 192 h after treatment, the foci level was very high. The colocalization level indicated the presence of up to 50 DSBs per cell in the senescent population (Figure 2C for LN229 and Figure 2D for A172). The sustained presence of DNA damage in senescent cells was confirmed by the neutral comet assay, which revealed a significant increase in DSBs in both LN229 (Figure 2E) and A172 cells (Figure 2F).

### 3.3. Are TMZ-Induced DSBs in CSEN Cells Localized in Telomeres?

**Figure 3:**
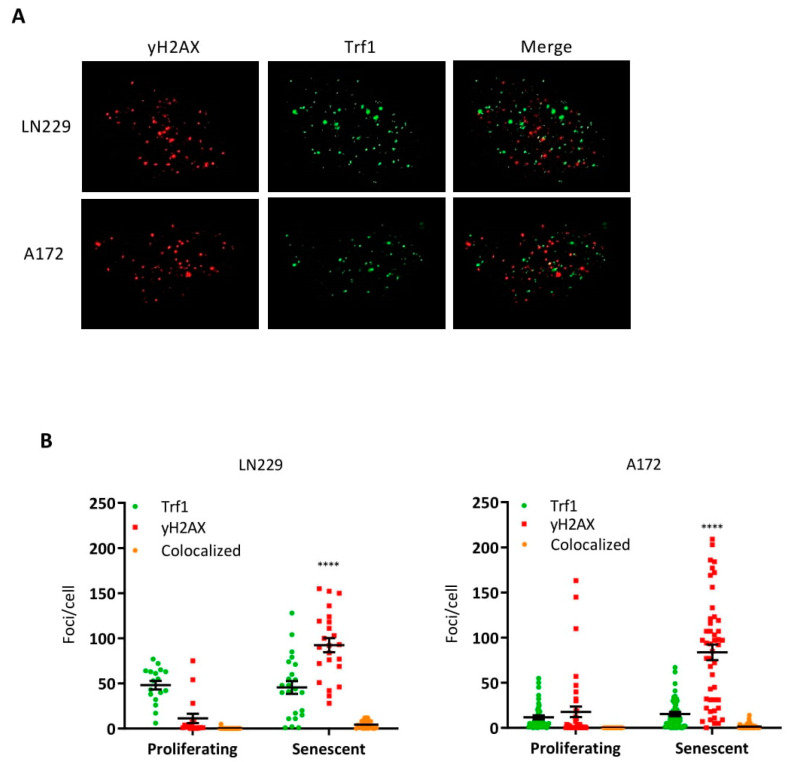
DNA double-strand breaks and telomeres labeled through Trf1 in TMZ-treated LN229 and A172 cells. (**A**) Representative images of γH2AX and Trf1 staining in senescent LN229 and A172 cells (8 days after TMZ treatment). (**B**) Quantification of Trf1 and γH2AX foci and their colocalization in proliferating and senescent LN229 and A172 cells. At least 50 cells were analyzed in each case. The difference in γH2AX foci between proliferating and senescent populations was highly significant, **** *p* < 0.0001.

DSBs in telomeres are considered to be repaired less effectively, thus contributing to the sustained damage response in senescent cells [30]. Therefore, we assessed the number of foci in telomeric regions by means of immunostaining of γH2AX and telomeric repeat binding factor (Trf1), which is a marker of telomeres [31]. Representative images are shown in Figure 3A. DSBs were indicated by γH2AX foci, while telomeres were visualized by Trf1 foci. As revealed by quantification and colocalization assessment, γH2AX foci did not colocalize with telomere foci (Figure 3B,C), indicating that O^6^MeG-induced DSBs in senescent cells are not preferentially localized in telomeres.

### 3.4. Are DSBs Subject to Repair in Senescent Cells?

**Figure 4:**
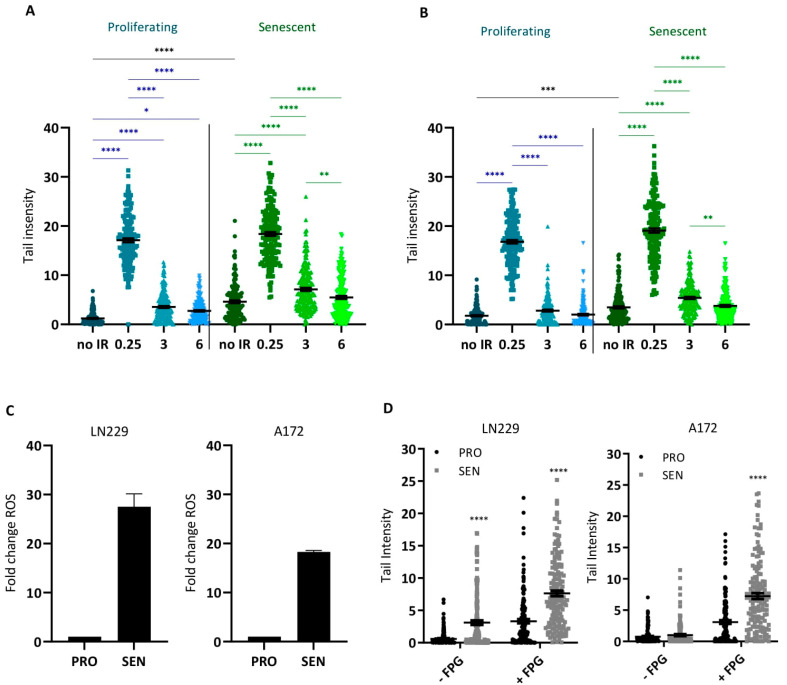
DSBs and oxidative stress in the proliferating and senescent cell population. (**A**,**B**) DSBs were measured immediately (0.25 h) after IR (6 Gy) and 3 and 6 h thereafter. Neutral comet assay of nonirradiated and irradiated proliferating and senescent LN229 (**A**) and A172 (**B**) cells. Bars indicate the median ± SEM of three independent experiments. For each experiment, 50 cells were analyzed. * *p* < 0.05, ** *p* < 0.01, *** *p* < 0.001, **** *p* < 0.0001. (**C**) ROS level in exponentially growing (control) and CSEN cells. (**D**) Alkaline comet assay in the absence and presence of FPG protein on proliferating and CSEN cells. The differences between control and CSEN were highly significant.

DSBs are quickly repaired by homologous recombination (HR) or, more slowly, by nonhomologous end-joining (NHEJ). To test if DSBs were repaired in senescent cells, we irradiated senescent and proliferating LN229 and A172 cells with 6 Gy and measured DSBs via the neutral comet assay immediately (0.25 h) and 3 and 6 h after irradiation. Non-irradiated proliferating and senescent cells served as controls. Senescent LN229 and A172 cells (not irradiated) showed higher tail intensities than proliferating cells (Figure 4A,B), supporting the data shown above that a high number of DSBs are present in senescent cells. Following irradiation, the number of DSBs increased in proliferating and senescent cells to the same extent, and 3 and 6 h after radiation treatment the DSB level returned to that of the non-treated control (Figure 4A,B). This data indicates that senescent glioblastoma cells are capable of DSB repair.

### 3.5. ROS and Oxidative DNA Damage in Senescent Cells

It was shown that senescent cells contain a high amount of ROS [32]. We determined the ROS level in exponentially growing (control) and TMZ-induced CSEN cells and observed that the ROS level was significantly enhanced in CSEN cells (Figure 4C). Concomitantly, the amount of ROS-induced DNA damage was enhanced, as proven by the alkaline comet assay that measures single-strand breaks, and the FPG-modified alkaline comet assay that additionally measures oxidative DNA lesions such as 8-oxo-guanine, which are recognized by the FPG protein [33]. In both LN229 and A172 cells, the number of FPG-positive sites was significantly enhanced (Figure 4D), indicating that cellular ROS induces oxidative DNA damage in senescent cells. At the same time, we observed an increase in the amounts of Nrf2 and hemoxygenase-1 (Figure S3), which are established cellular indicators for ROS-provoked cellular stress [34].

### 3.6. Is O^6^MeG Required for Maintaining the Senescent State?

**Figure 5:**
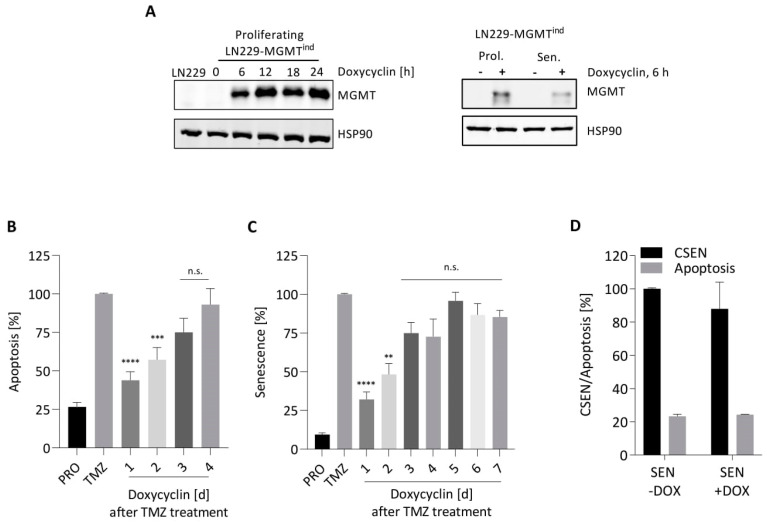
O^6^MeG is required for induction of apoptosis and CSEN, but not for maintenance of the senescent state. (**A**) Proliferating and senescent LN229-MGMT^ind^ cells show strong induction of MGMT following doxycycline treatment. (**B**,**C**) LN229-MGMT^ind^ cells were treated with 50 µM TMZ and 100 ng/mL doxycycline for the indicated times, and apoptosis (**B**) and CSEN (**C**) were measured 5 and 10 days following TMZ treatment, respectively. (**D**) The senescent population obtained 8 days after TMZ treatment (50 µM) was not treated (CSEN) or treated with doxycycline; 48 h later, apoptosis and CSEN were measured by flow cytometry. Induction of MGMT had no impact on their levels. Data are the mean of three independent experiments ± SEM. ** *p* < 0.01, *** *p* < 0.001; **** *p* < 0.0001; n.s. = not significant.

The alkylation lesion O^6^MeG is a potent inducer of senescence [7]. Is the presence of this damage also necessary for maintaining the senescent phenotype? To provide an answer to this question, we used LN229 cells harboring MGMT cDNA under control of doxycycline (Dox). Following addition of Dox to the medium, the MGMT protein was expressed within 6 h in both proliferating and senescent cells (Figure 5A). If exponentially growing cells were treated with TMZ followed by Dox treatment at different times thereafter (in 24 h intervals, 24–96 h), the yield of dead cells (mostly apoptosis) increased successively. Thus, switching on MGMT expression 1 and 2 days after TMZ significantly reduced the cell death level, while MGMT expression 3 days after TMZ and later was ineffective in causing cell death protection (Figure 5B). Obviously, repair of O^6^MeG lesions in the early period (2–3 days) after damage induction is important for the killing defense.

We performed similar experiments with CSEN as the endpoint. The level of senescent cells was significantly reduced if MGMT was induced by Dox within a 2 day period after TMZ exposure. Thereafter, MGMT expression no longer had a significant effect on the induced senescence level (Figure 5C). This supports the notion that O^6^MeG is the trigger of TMZ-induced cell death and senescence [7], and that conversion of O^6^MeG into the critical downstream lesion (DSBs) and activation of the apoptotic and senescent cascade utilize the same pathway, which takes several days. Interestingly, MGMT upregulation by Dox in senescent cells had no impact on their survival; the senescence and apoptosis level remained unaffected (Figure 5D). Overall, the data revealed that O^6^MeG is required for the induction of senescence, but not for maintenance of the senescent state.

### 3.7. Sustained Activation of the DNA Damage Response in Senescent Cells

**Figure 6:**
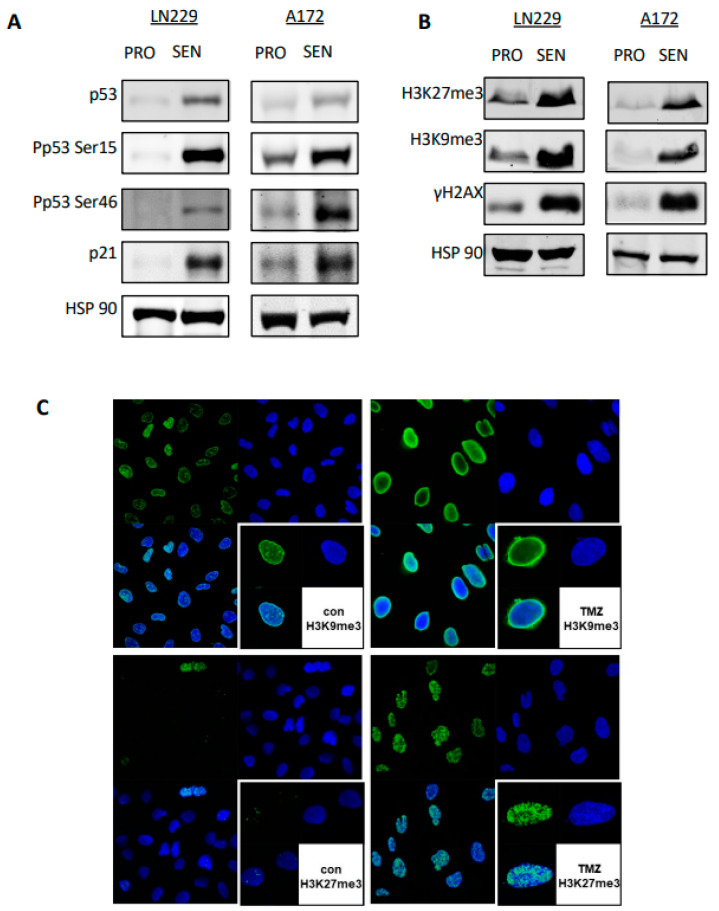
Senescent cells show a sustained DDR and senescence-associated histone methylation. (**A**) Expression levels of p53, phosphorylated p53ser15 and ser46, and p21 in proliferating (PRO) and TMZ-induced senescent LN229 and A172 cells. (**B**) Levels of marker of senescence in proliferating and TMZ-induced senescent LN229 and A172 cells. (**C**) Immunohistochemistry of nontreated and TMZ-treated (50 µM, 144 h post exposure) LN229 cells with senescence-associated histone methylation marker H3K9me3 and H3K27me3. Green fluorescence: histone marker; blue: nuclear staining with topo3; white: merged staining. Right bottom corner: field with higher magnification.

The presence of DSBs in TMZ-induced senescent cells led us to predict that the DDR is permanently activated. WB analysis indicated that this is actually the case. Thus, we observed a clear increase in the level of total p53, p53Ser15, p53Ser46, and p21, which is a target gene of activated p53 (Figure 6A). We also observed an increase in methylated histones H3K27me3 and H3K9me3, as well as γH2AX, all of which are hallmarks of DDR (Figure 6B). The high amount of H3K27me3 and H3K9me3 protein in CSEN cells was confirmed by immunocytochemistry (Figure 6C). The data indicate that chromatin conformational changes did occur in CSEN cells. In contrast, replication-dependent DNA repair proteins (MSH2, MSH6, RAD51) were downregulated in TMZ-induced CSEN (Figure S4), confirming a previous report [18].

### 3.8. Is Acquired TMZ Resistance Associated with Resistance to Induction of CSEN?

**Figure 7:**
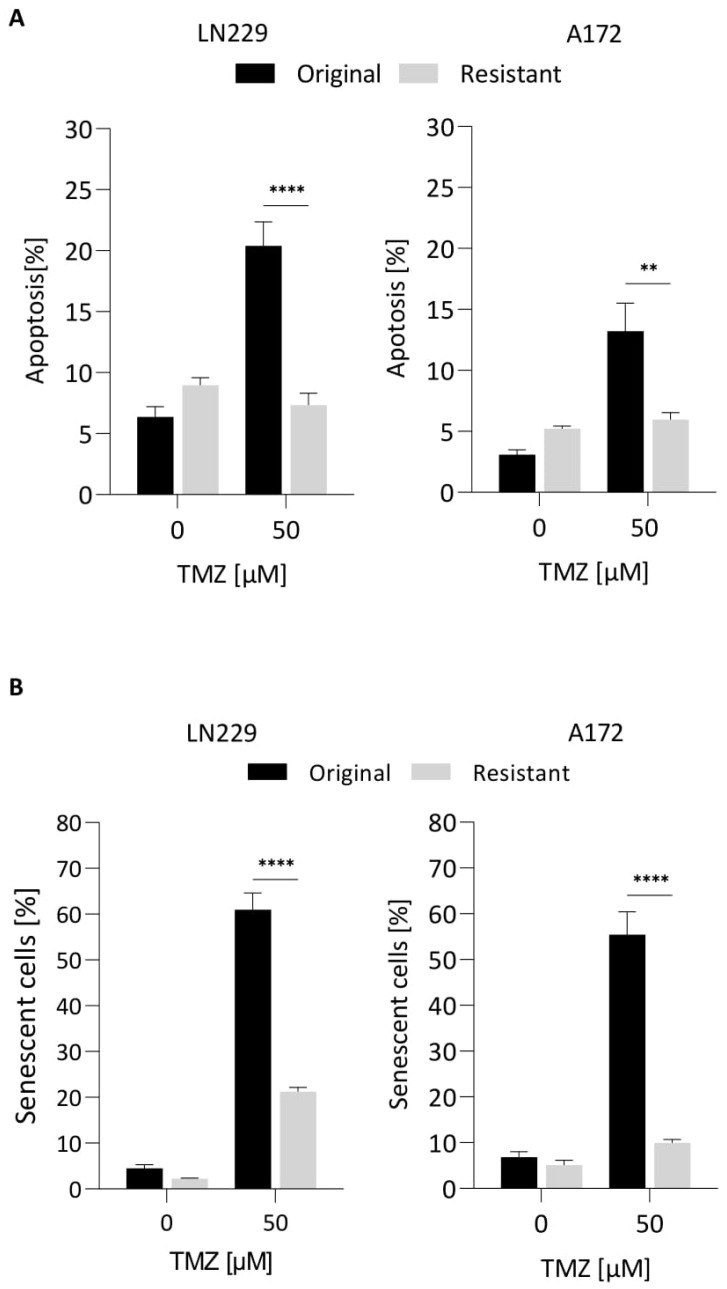
Repeated TMZ treatment leads to TMZ resistance of LN229 and A172 cells. LN229 and A172 cells were repeatedly treated with 50 µM TMZ. Surviving cells were reseeded and treated with 50 µM TMZ; apoptosis (**A**) and CSEN (**B**) were measured 5 and 10 days later, respectively. TMZ resistance is indicated by a lower induction of apoptosis and CSEN. ** *p* < 0.01, **** *p* < 0.0001.

Repeated treatment of glioma cells with TMZ leads to resistance to the cytotoxic effect of TMZ. It is unclear, however, whether this also applies to CSEN. We addressed this question by treating LN229 and A172 cells repeatedly with TMZ and selected for survivors. The surviving population was assessed for their ability to undergo apoptosis and CSEN. Following treatment with 50 µM TMZ, significantly fewer cells died by apoptosis in the resistant population (Figure 7A). The same was true for CSEN; significantly fewer cells were arrested in the senescent state (Figure 7B). We should note that the resistant population was not characterized by an increase in the MGMT level (Figure S5), indicating that acquired resistance in the presence of O^6^MeG results in protection against both cell death and senescence.

### 3.9. TMZ-Induced CSEN Displays the Inflammatory Phenotype

A hallmark of CSEN is the senescence-associated secretory phenotype (SASP), which is characterized by secretion of proinflammatory cytokines such as IL-1 and IL-6. We measured the expression of proinflammatory cytokines on an RNA level and show that, in the TMZ-induced senescent population, IL-1A, IL-1B, IL-6, and IL8, as well as CCL2, CCL8, and CXCL1, were strongly upregulated in LN229 and A172 cells (Figure S6). This confirms our previous observation that CSEN upon TMZ treatment is associated with SASP [18].

### 3.10. Senescence in Glioblastoma In Situ Following TMZ-Based Therapy

**Figure 8:**
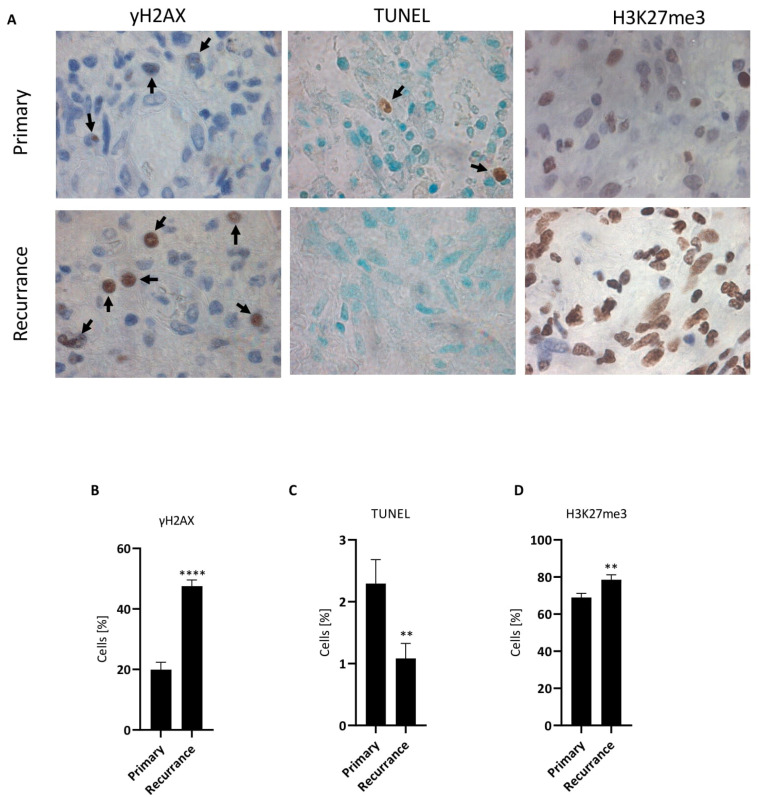
Immunostaining with γH2AX, TUNEL, and H3K27me3 in tumor sections of primary GBM and recurrences. (**A**) Representative images of γH2AX and TUNEL staining. (**B**) Quantification of γH2AX staining (% positive cells), (**C**) TUNEL-positive and (**D**) H3K27me3-positive cells. In all cases, the difference between primary tumor and recurrence was highly significant (** *p* < 0.01, **** *p* < 0.0001).

Cellular senescence relies on the induction of a molecular pathway that represents, like apoptosis, a basic cellular entity. It is, therefore, likely that CSEN is also induced in cancer cells in vivo during chemotherapy. Evidence, however, is still lacking for GBM. We studied this by staining GBM specimens and comparing primary tumor specimens with the corresponding recurrent tumor from patients treated with TMZ. In recurrent tumors, we found a significantly higher number of cancer cells that were positive for γH2AX (see Figure 8A for representative images and Figure 8B for quantification). This indicates that, in recurrent tumors, DSBs, a hallmark of TMZ-induced senescence, were accumulating. We measured at the same time the proportion of apoptotic cells by TUNEL staining (see Figure 8A for representative images) and found it to be reduced in recurrent tumors (Figure 8C). This indicates that, in recurrent tumors, senescent cells accumulated and prevented cells from undergoing apoptotic death. As shown in Figure 6B, the methylated histone H3K27me3 was enhanced following TMZ treatment in senescent cells, supporting the view that it is a senescence marker [35]. Therefore, we also stained paraffin-embedded primary and recurrent specimens with H3K27me3. The staining intensity of recurrent cancer cells was clearly higher (Figure 8A), and a significantly higher proportion of glioblastoma cells were positively stained with this marker (Figure 8D). In summary, the data support the notion that in recurrent GBM, following radio-chemotherapy and adjuvant TMZ, senescent cancer cells are present at a higher amount than in the primary tumor.

## 4. Discussion

TMZ, first-line drug in the treatment of GBM, is effective if the tumor lacks MGMT or expresses the repair protein at a very low level, i.e., <30 fmol/mg protein [36]. These tumors have been shown to be MGMT promoter-methylated [9,37]. Although the improved overall survival is significant, the prognosis of GBM is still bleak with a median survival of only 14.6 months (12.6 and 23.4 months in the MGMT-unmethylated and MGMT-methylated subgroups, respectively) [38]. Although recent clinical trials indicated a trending increase in survival, e.g., by including nitrosoureas such as lomustine [39], the prognosis is still bad with a 5 year overall survival of less than 10% [40]. Even though the mechanism of TMZ is well described [19,41], it remains a mystery why GBM responds so poorly to radio-chemotherapy. A commonly held view is that GBM rapidly develops radiation and drug resistance.

Previously, we showed that TMZ induces not only apoptosis, but also CSEN [7,18]. Both effects are triggered by O^6^MeG, and the presence of MGMT and lack of MMR prevent apoptosis and CSEN, indicating that the upstream DNA damage response pathways are identical [7]. In the present work, we studied CSEN induced by TMZ in detail, showing that cells enter the senescence phase late after treatment with a time course that parallels apoptosis. However, CSEN occurs at an about fourfold higher level than apoptosis and, therefore, appears to be the major response induced by TMZ.

O^6^MeG itself is not toxic. It is converted, however, into DSBs by futile MMR, which occurs in the post-treatment cell cycle [15]. Here, we demonstrate that, in GBM cells, DSBs show a time course that parallels apoptosis and CSEN. Analysis of the senescent population (>6 days after TMZ treatment) revealed a significantly increased level of DSBs in CSEN. These DSBs were not colocalized with Trf2, the shelterin protein repeat telomeric binding factor, which is localized on telomeres [42]. We stress this point as it has been shown that DSBs in telomeres, which are not subject to repair can trigger drug-induced senescence [43]. We hypothesize that O^6^MeG/MMR-mediated DSBs occur randomly in the genome. Basically, they can be repaired; however, if some breaks are not accessible to repair, they persist and provide a constant trigger for activating the DDR. Actually, Western blot analysis showed increased CHK1 and CHK2 activation, as well as p53Ser15/Ser46 phosphorylation, indicating p53 activation, which was confirmed by upregulation of the target p21. We also showed increased trimethylation at H3K27 and H3K9, which are hallmarks of CSEN [35]. Interestingly, TMZ-induced senescent cells were also characterized by a high amount of intracellular ROS that contributed to DNA damage, as revealed by high single-strand break (SSB) and 8-oxo-guanine levels in senescent cells. Theoretically, DSBs could result from this intracellular ROS. This, however, is unlikely since SSBs and replication blocking base damages need replication in order to be converted into DSBs, while senescent cells do not replicate.

The increased DSB level in senescent cells raises the question of whether senescent cells are capable of DNA repair. Therefore, we irradiated CSEN cells and measured DSB repair by means of the neutral comet assay. A significant reduction in tail intensity 3 and 6 h post irradiation indicated that senescent cells are capable of DNA damage repair. If senescent cells are DSB repair-competent, why are DSBs still present? Can residual O^6^MeG in senescent cells be a source for DSBs? To answer this question, we treated senescent LN229 cells that harbor an inducible MGMT vector (LN229-MGMT^ind^) with doxycycline to induce MGMT expression. We did not observe an impact on the senescent cell level or an increase in apoptosis in this population, indicating that O^6^MeG is not required for maintaining the senescent state. Since senescent cells are able to repair DSBs, we favor the hypothesis that DSBs originated in the CSEN induction phase from O^6^MeG lesions in a process described above. Some of these DSBs, which arrived a plateau 72 h after treatment, remained unrepaired. These residual DSBs are obviously key in maintaining the senescent state through permanent activation of the DDR.

DSBs also trigger the apoptotic pathway. Dose-response studies showed that apoptosis and CSEN increased linearly with dose in p53 wild-type GBM cells, and neither endpoint displayed a significant threshold [23,26]. For LN229 cells, we determined a relation of 950 and 410 O^6^MeG adducts per 1% increase in the level of apoptosis and senescence, respectively. For A172 cells, the relationship was 1580 and 440 O^6^MeG per 1% increase in cell death and senescence, respectively [23]. Thus, although O^6^MeG is able to activate both pathways, the CSEN pathway is more susceptible to activation than the apoptosis pathway. We are aware of the limitation of our work as we assessed these correlations in only two GBM cell lines, which are functionally wild-type for p53. Further studies are required, including cells mutant or deficient for p53.

Using an inducible MGMT vector system, we also addressed the question of how long DSBs have to be present in order to induce the responses. Thus, we induced MGMT by doxycycline for 1 to 8 days following TMZ treatment. If MGMT was upregulated immediately after treatment, apoptosis and CSEN were completely abrogated. In contrast, when induction of MGMT occurred later than 3 days following treatment, no significant reduction in senescence and apoptosis was observed. This finding indicates that the conversion of O^6^MeG into DSBs reaches a critical point 3 days following TMZ treatment, leading to an activation of the pathways.

Repeated treatment with TMZ leads to a cell population almost completely composed of senescent cells. However, after weeks of cultivation, islands of surviving cells grow out and formed mini-colonies. These cells gave rise to a new cell population, which grew similarly to the parental cells. Treatment of this surviving population showed that they gained resistance not only to the toxic effect of TMZ (apoptosis), but also to CSEN. We did not characterize the resistant cell clones in detail, which was reported in another study [44], but the data suggest that the mechanism leading to drug resistance impacts both the senescence and the apoptosis pathway. This is expected if acquired drug resistance relies on upregulation of MGMT or downregulation of MMR or DSB repair [44], which are upstream of both pathways. We should note that we did not observe reactivation of MGMT in our cell populations that acquired TMZ resistance.

Taken together, our results show that, following TMZ treatment, senescence and apoptosis are induced simultaneously in the population, but senescence is the main trait (as indicated by increased SA-β-galactosidase, increased C12FDG, increased DSBs, sustained DDR, increased polyploidy level and histone methylation, and gain of SASP hallmarked by upregulation of key inflammatory genes). We should stress the point that the TMZ concentration used in our experiments (up to 50 µM) is in the therapeutic range. Thus, the serum concentration of TMZ has been determined to be in the range of 20–70 µM [1,41,45,46,47,48], and, with a single oral dose of 150 mg/m^2^, the peak plasma concentration and the brain interstitium concentration were 28.4 and 1.5 µM, respectively [49]. Following oral 200 mg/m^2^ TMZ, the plasma peak level was 72 µM and the concentration in the cerebrospinal fluid was 9.9 µM [50]. It is important to note that TMZ is administered daily, and, in a respective experimental setting, repeated low daily doses gave rise to significant accumulation of DSBs, apoptosis, and CSEN [51]. Therefore, we consider that the results obtained in a clinically relevant dose range are therapeutically relevant.

We should also note that, in the therapeutic situation, radiation and TMZ are given concurrently, while in the subsequent maintenance therapy TMZ is administered alone. In this study, we used TMZ only. The interaction of radiation and TMZ, notably the induced critical lesions, is surely an attractive area of research, which may be stimulated by this work. We are aware of the limitation of this study with GBM cell lines. However, we see no reason to assume that the activation of apoptosis and CSEN pathways triggered by O^6^MeG adducts does not also take place in GBM cells embedded in a complex tissue structure in vivo, since these reflect basic molecular processes. The detection of senescence markers in recurrences (Figure 8) can be viewed as supportive in this direction.

## 5. Conclusions and Hypotheses

What are the inferences of the findings regarding therapy? The data suggest that, after surgical removal of the primary tumor, the residual tumor cells can only partially be killed by subsequent TMZ therapy. The majority of cells remain in a dormant state as a senescent population. Consequently, the recurrent tumor is expected to contain a large number of senescent cells. This was actually shown by a comparison of primary tumor and recurrence (paired samples), which revealed a significantly higher number of cells positively stained with γH2AX-displaying foci. Control TUNEL staining, which is indicative of apoptotic cells, did not reveal a difference between primary tumor and recurrence, supporting the view that the recurrences contain a large fraction of senescent cells or cells escaping senescence with a significant amount of residual DNA damage. It is clear that this requires confirmation on a larger tumor panel and additional senescence markers.

The high number of senescent cells may appear ideal at first glance, but problems arise on closer inspection. Firstly, no one knows how long the senescent cells are dormant. Although CSEN is defined as an irreversible cessation of proliferation, there is increasing evidence that senescent cells can become capable of proliferation. Secondly, CSEN is characterized by the inflammatory phenotype, as also shown for TMZ-induced CSEN [18]. This, however, is associated with a negative prognosis, since proinflammatory cytokines produce a tumor environment that promotes tumor growth. In conclusion, it is important not only to kill proliferating cancer cells, but also to eliminate the senescent cell population. This might be achieved through the use of senolytic agents administered after the first cycles of TMZ treatment or in the recurrent state. Potent senolytic agents have been identified [52], and a search for GBM-specific senolytics is in progress in our laboratory.

## Data Availability

The data presented in this study are available in this article.

## Supplementary Materials

The following supporting information can be downloaded at https://www.mdpi.com/article/10.3390/cancers14092233/s1. Figure S1: Induction of senescence and cell death following TMZ treatment. (A) Representative images of proliferating and senescent LN229 and A172 cells with and without X-gal staining. (B) Quantification of early apoptosis, late apoptosis/necrosis and total cell death following treatment of LN229 and A172 cells with 50 μM TMZ. Figure S2: Comparison of x-gal staining with manual analysis using microscopy and flow cytometric analysis following C12FDG staining. Figure S3: WB analysis of proliferating (PRO) and senescent (SEN) LN229 and A172 cells showing increased ROS markers NRF2 and hemoxygenase 1. HSP90 served as loading control. Figure S4: Senescent (SEN) cells show reduced DNA repair markers compared to proliferating (PRO) cells. Figure S5: TMZ resistance is not due to upregulation of MGMT. WB analysis of resistant and parental non-resistant LN229 and A172 cells, which do not display MGMT protein. Dox treated MGMTind cells served as positive control. Figure S6: Comparison of expression of SASP markers in the proliferating and TMZ-induced senescent cell population. (A), LN229; (B) A172 cells. Quantitative RT-PCR analysis. The raw data for western blots are available as Supplement: Raw data.
