# Supplementary material for: Senescence Is the Main Trait Induced by Temozolomide in Glioblastoma Cells
Source: Cancers (Basel). 2022 Apr 29;14(9):2233. doi: 10.3390/cancers14092233 (PMC9102829; doi:10.3390/cancers14092233)
Supplement: Supplementary file 1 [file cancers-14-02233-s001.zip › cancers-1663198 supplementary for xml.pdf]

cancers MDPI

# Supplementary Materials: Senescence Is the Main Trait Induced by Temozolomide in Glioblastoma Cells

**Lea Beltzig, Christian Schwarzenbach, Petra Leukel, Katrin B. M. Frauenknecht, Clemens Sommer, Alessandro Tancredi, Monika E. Hegi, Markus Christmann and Bernd Kaina***

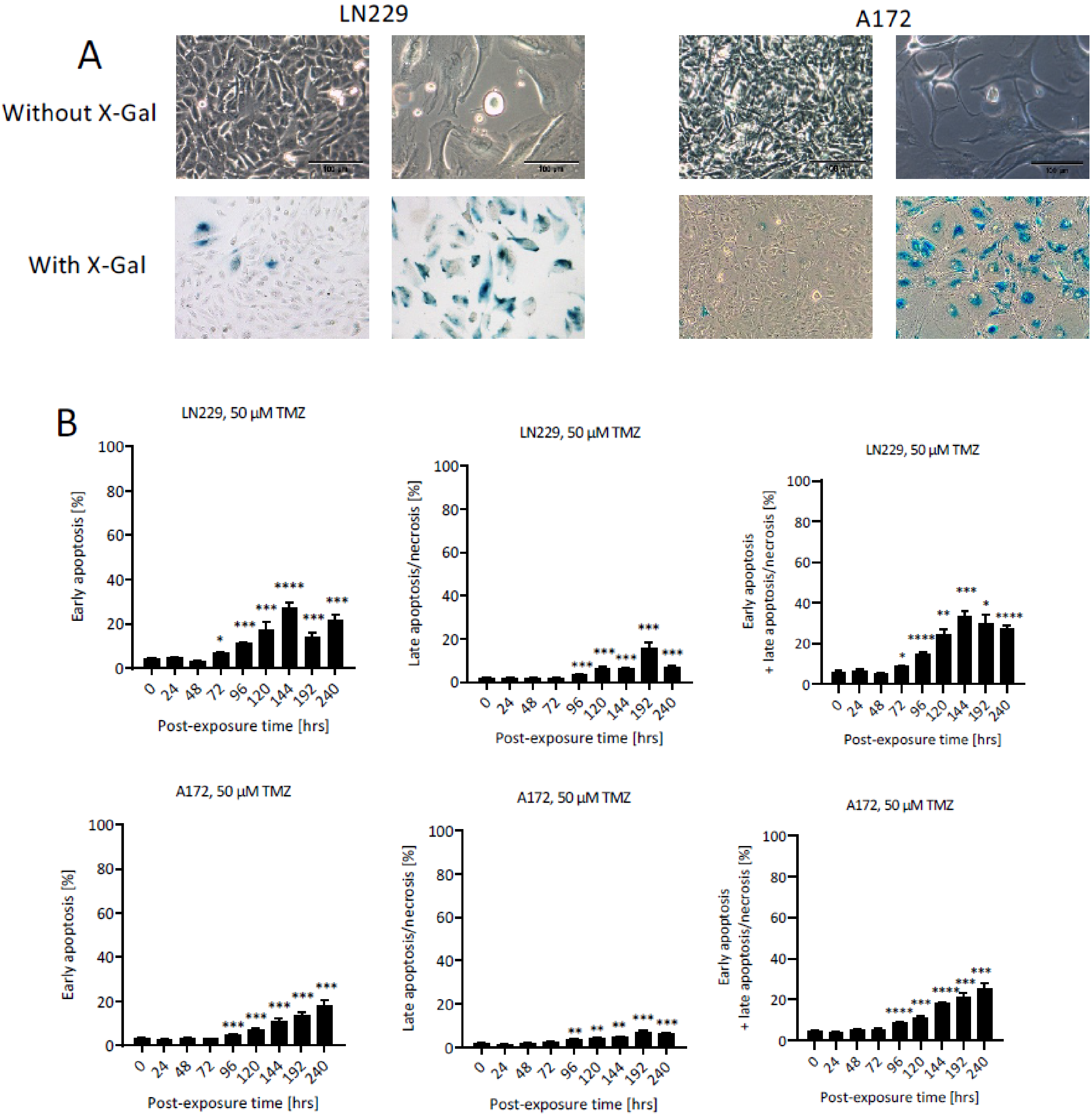


**Figure S1.** Induction of senescence and cell death following TMZ treatment. (A) Representative images of proliferating and senescent LN229 and A172 cells with and without X-gal staining. (B) Quantification of early apoptosis, late apoptosis/necrosis and total cell death following treatment of LN229 and A172 cells with 50 µM TMZ. * $p < 0.05$, ** $p < 0.01$, *** $p < 0.001$; **** $p < 0.0001$.

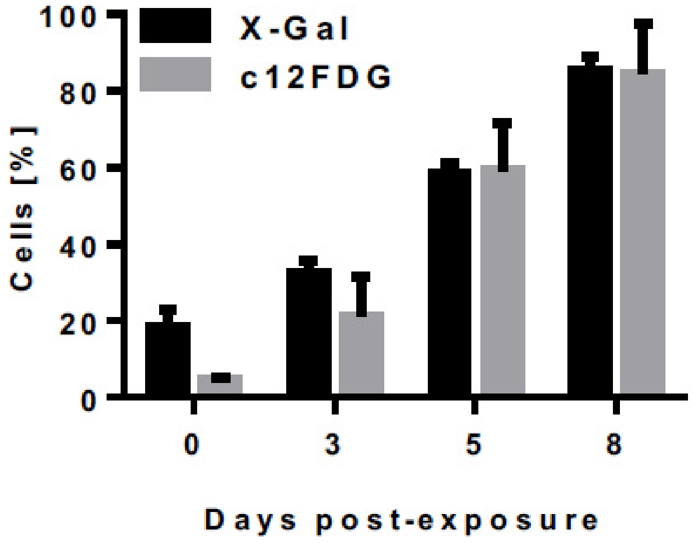


**Figure S2.** Comparison of x-gal staining with manual analysis using microscopy and flow cytometric analysis following C12FDG staining.

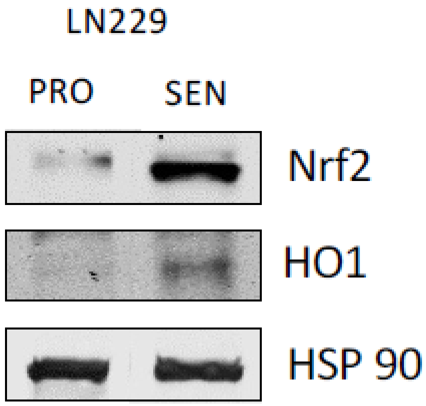


**Figure S3.** WB analysis of proliferating (PRO) and senescent (SEN) LN229 and A172 cells showing increased ROS markers NRF2 and hemoxygenase 1. HSP90 served as loading control.

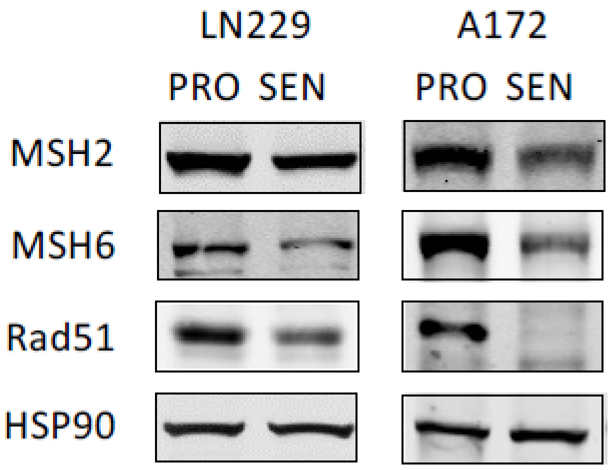


**Figure S4.** Senescent (SEN) cells show reduced DNA repair markers compared to proliferating (PRO) cells.

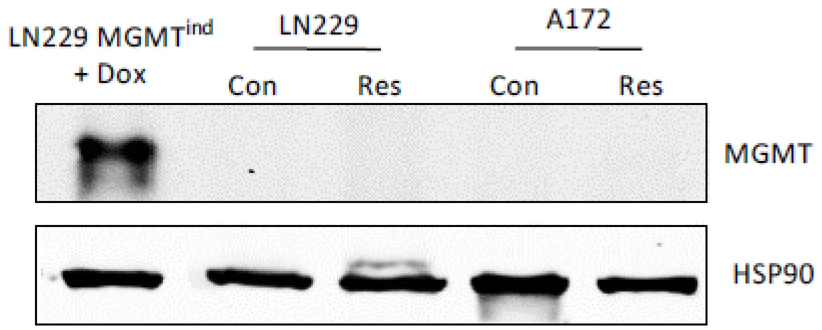


**Figure S5.** TMZ resistance is not due to upregulation of MGMT. WB analysis of resistant and parental non-resistant LN229 and A172 cells, which do not display MGMT protein. Dox treated MGMTind cells served as positive control.

A

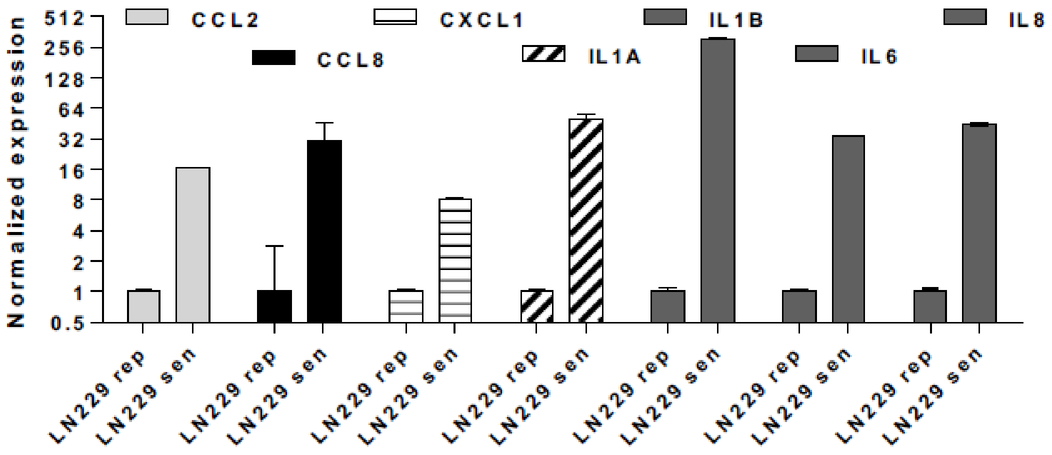


B

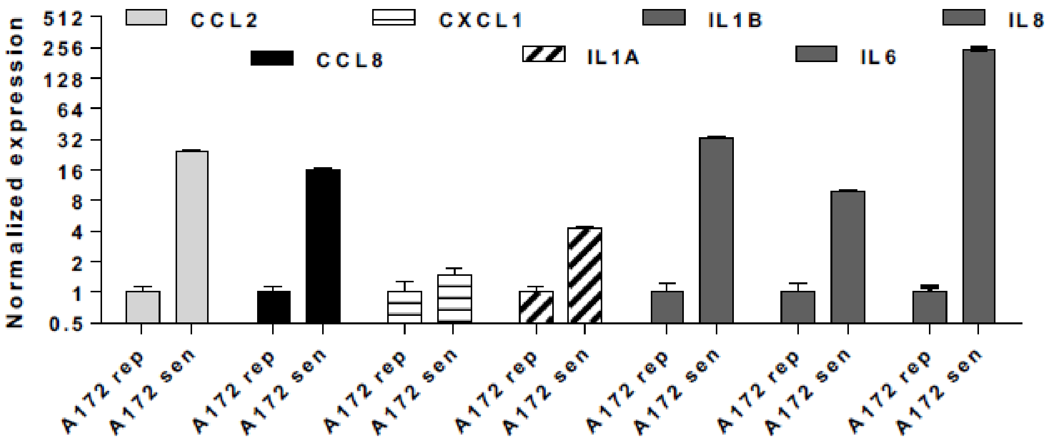


**Figure S6.** Comparison of expression of SAIP markers in the proliferating and TMZ-induced senescent cell population. (A), LN229; (B) A172 cells. Quantitative RT-PCR analysis.
