# Supplementary material for: Senescence Is the Main Trait Induced by Temozolomide in Glioblastoma Cells
Source: Cancers (Basel). 2022 Apr 29;14(9):2233. doi: 10.3390/cancers14092233 (PMC9102829; doi:10.3390/cancers14092233)
Supplement: Supplementary file 1 [file cancers-14-02233-s001.zip › cancers-1663198 supplementary Raw data for xml.pdf]

cancers

MDPI

Supplementary Material: Senescence Is the Main Trait Induced by Temozolomide in Glioblastoma Cells

Lea Beltzig, Christian Schwarzenbach, Petra Leukel, Katrin B. M. Frauenknecht, Clemens Sommer, Alessandro Tancredi, Monika E. Hegi, Markus Christmann and Bernd Kaina*

Supplement: Raw Data

Blot for kinetics of MGMT indution following Dox treatment
→ **Raw Data for Fig.5**

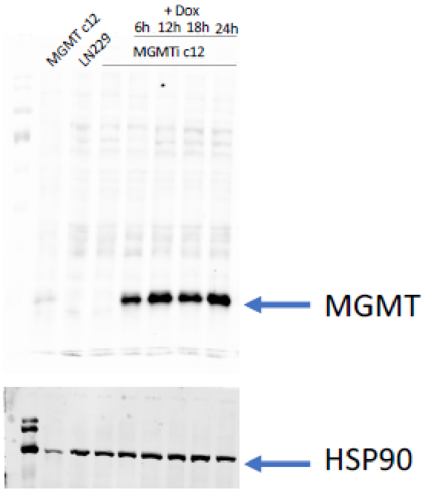

Blot for MGMT induction in replicating and senescent MGMTi
→ **Raw Data for Fig.5**

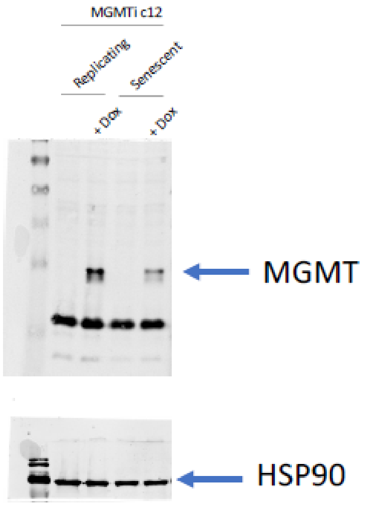

Raw data for Figure 5.

Blots for DDR
→ Raw Data for Fig.6 A

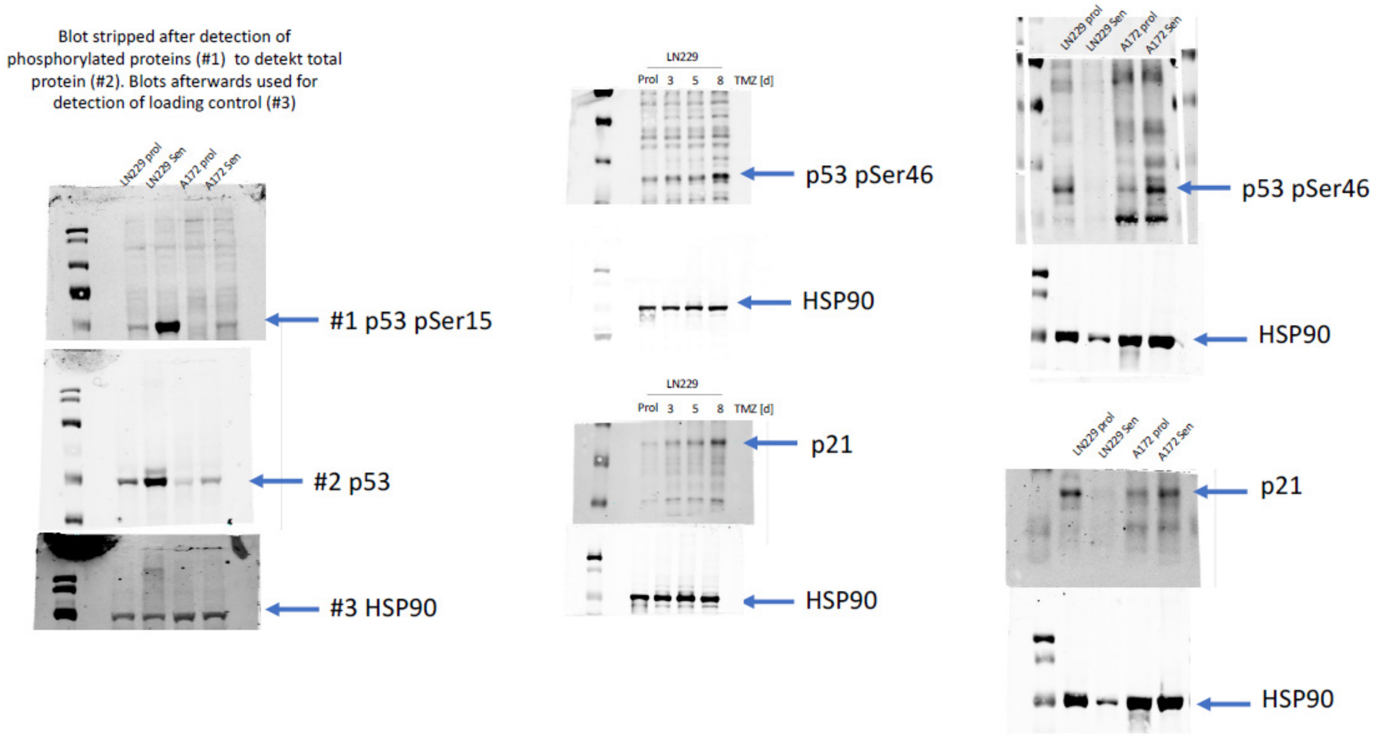

Raw data for Figure 6A.

Blot for DDR
→ Raw Data for Fig.6 B

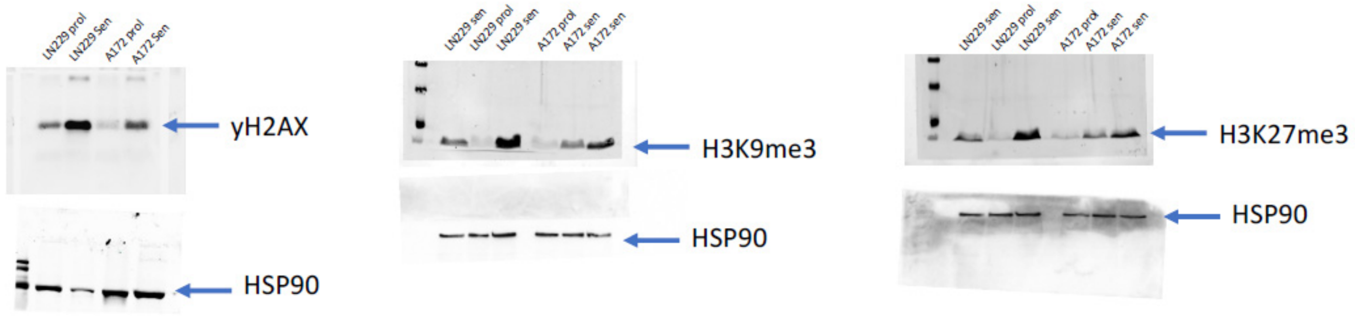

Raw data for Figure 6B.

Blot for DDR
→ Raw Data for Fig.S3

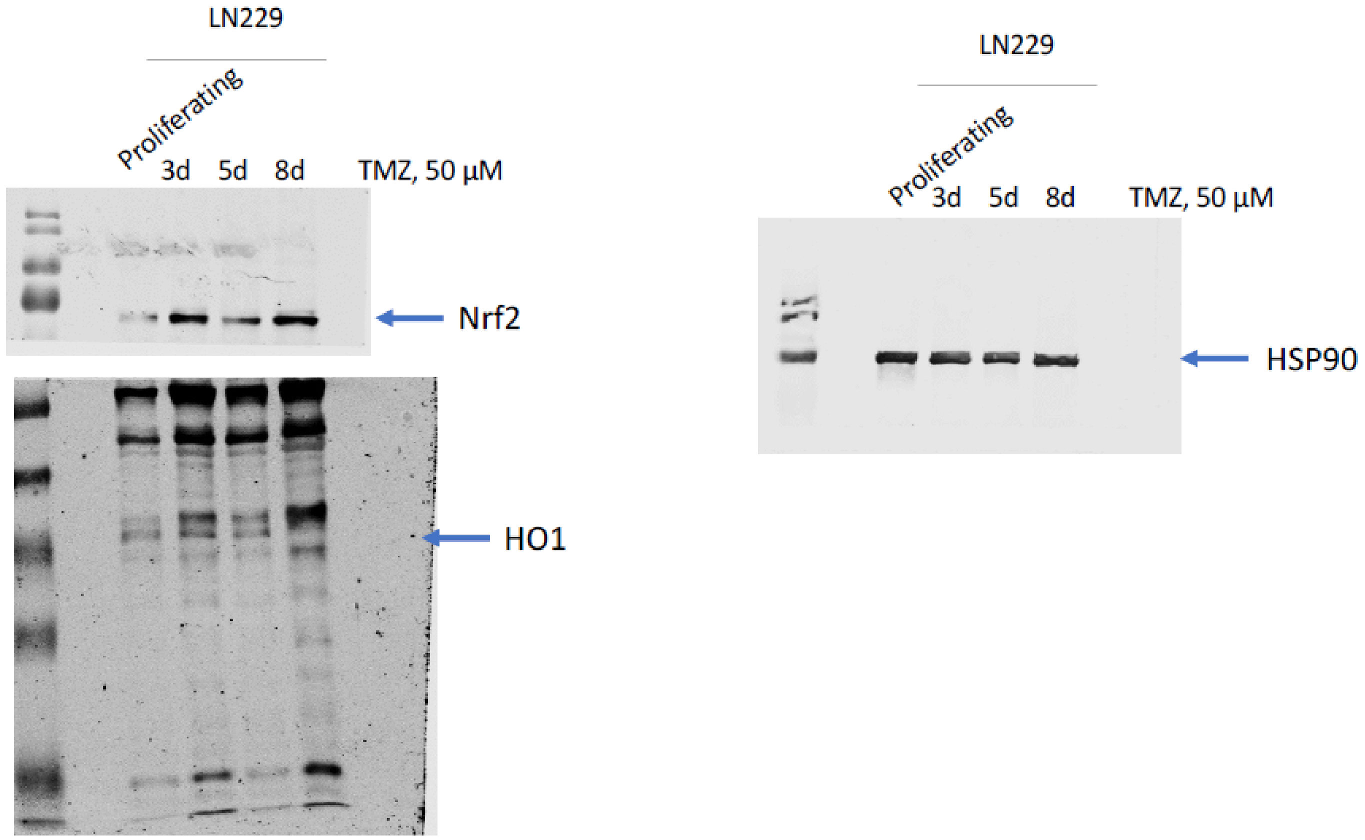

Raw data for Figure S3.

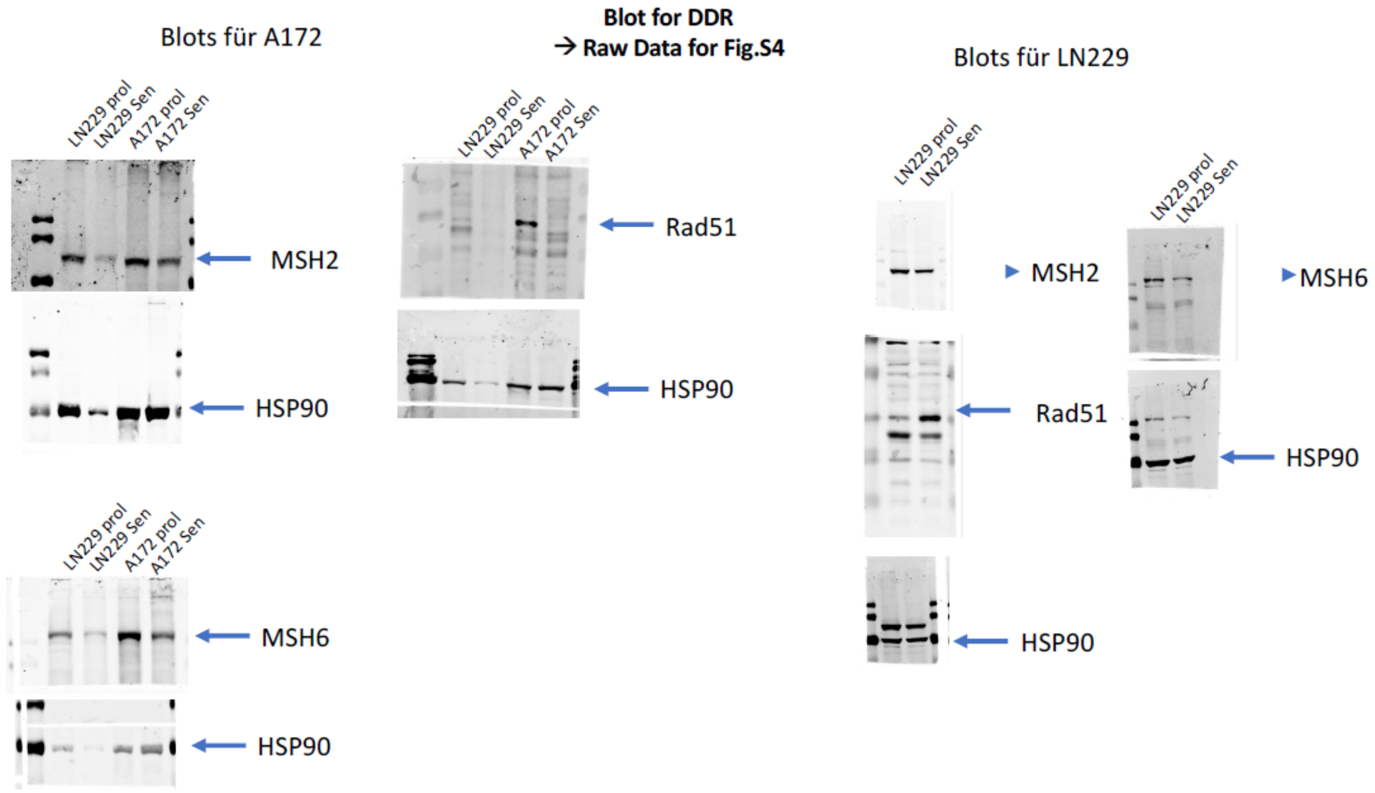

Raw data for Figure S4.

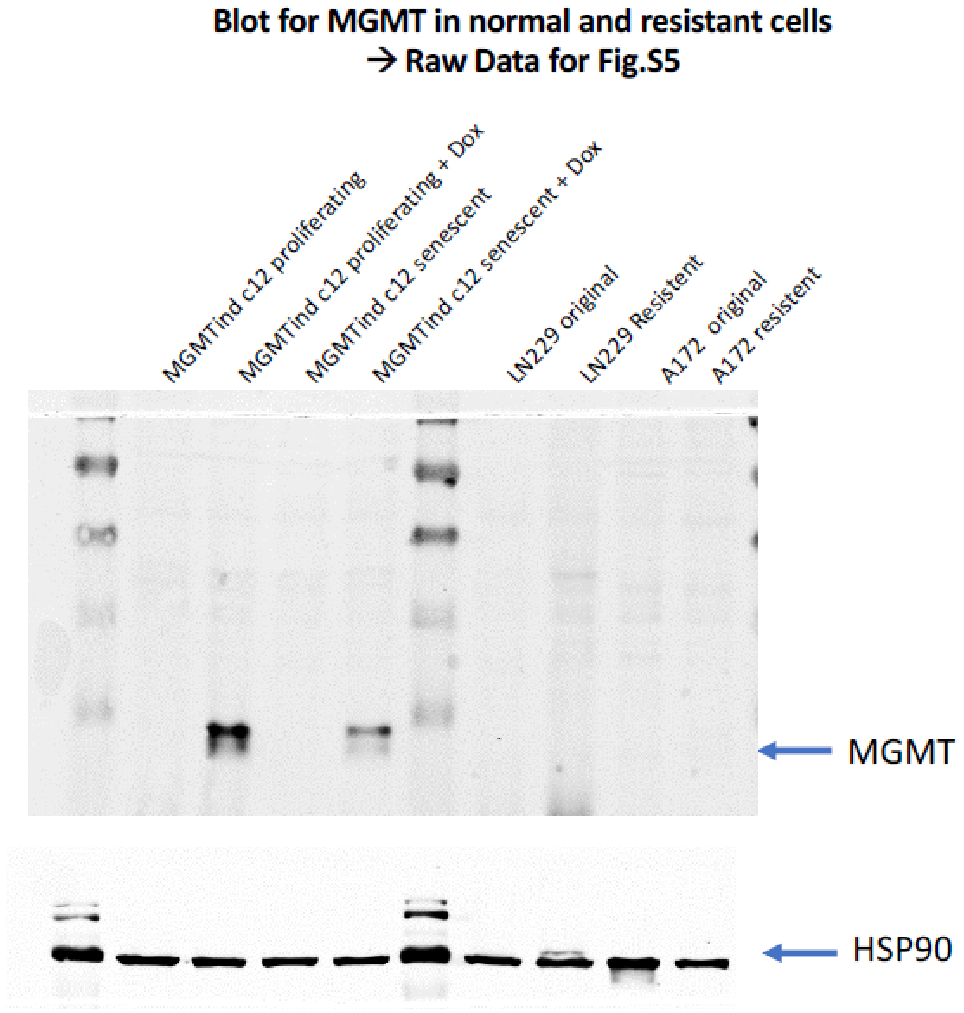

Raw data for Figure S5.
